# Long-term effect of soil and water conservation measures on runoff, sediment and their relationship in an orchard on sloping red soil of southern China

**DOI:** 10.1371/journal.pone.0203669

**Published:** 2018-09-07

**Authors:** Anguo Tu, Songhua Xie, Zhongbo Yu, Ying Li, Xiaofei Nie

**Affiliations:** 1 College of Hydrology and Water Resources, Hohai University, Nanjing, China; 2 Jiangxi Provincial Key Laboratory of Soil Erosion and Prevention, Nanchang, China; University of Waterloo, CANADA

## Abstract

The effect of soil and water conservation measures (SWCMs) is usually dependent on time. Thus the trend in reducing runoff and sediment over time is a very important theoretical problem for evaluating the effectiveness of SWCMs. Moreover, there is still a lack of comprehensive assessment of water erosion dynamics following implementing SWCMs despite their ecological significance. Therefore, the long-term impact of SWCMs on runoff and sediment and their relationships was assessed for an orchard on sloping red soil in southern China. Overland flow and erosion sediment were continuously observed for 15 years on citrus experimental plots under one of four treatments: grass strips, strip intercropping, level terrace and clean-tillage. By means of Mann–Kendall trend tests and double cumulative curves, the time series of runoff and sediment under the different treatments were analyzed. Furthermore, we linked the effect of soil conservation and the relationship between runoff and sediment variation to determine the mechanism of conservation measures on sediment reduction. The results showed that the first 4 years was the key period to prevent soil erosion for this orchard, and then the intensity of soil erosion decreased below 500 t·km^–2^·a^–1^. Considering economic costs and ecological effect, grass strips were the best protective measure for this test situation. The fitted curves of the effect of SWCMs on sediment reduction over time showed an ‘L’ form, but on runoff there was an approximately horizontal line. The SWCMs did not change the rainfall–runoff relationship, but did change the runoff–sediment erosion relationship. The erosion reduction mechanism of SWCMs in the early phase was a joint function of reducing runoff and changing the runoff–sediment relationship, and in the post-stable phase it worked mainly by reducing runoff. The results provide the basis for rational allocation of SWCMs considering location and time.

## Introduction

In recent decades, due to the effects of climate change, the increase in extreme precipitation events and human activities, there have been significant alterations in the process of runoff and suspended sediment in many rivers [1–4]. Significant decreasing trends in river sediment loads have been observed in approximately 50% of the world’s rivers [5, 6]. The application of integrated soil and water conservation measures (SWCMs) in watersheds is considered one of the main factors responsible for the reduced sediment discharge in river basins [7, 8]. Chu et al. [9]estimated that SWCMs accounted for 23% of the total sediment reduction by anthropogenic activities for nine major Chinese rivers during 1959–2007. Wang et al. [10] found that, from the 1990s onwards, SWCMs concerning vegetation were the main contributors to sediment reduction, accounting for 57% in the Yellow River. In the Mississippi River, vegetation restoration and soil conservation measures were the main causes for the reduction of suspended sediment[11, 12]. Long-term assessments of SWCMs on runoff and sediment change mostly use the watershed scale as the research object, but the dynamic effects with time of SWCMs on runoff and sediment are poorly understood. Moreover, because of the heterogeneity and complexity of the underlying surface of the basin, accurate identification of individual conservation measure effects on watershed-scale runoff discharge and sediment yield is often difficult [13], and how SWCMs affect the hydrological process and sediment transport has not been scientifically determined. For example, the question as to whether the sediment reduction mechanism of various SWCMs is due to reducing water runoff or changing the water–sediment relationship still has no definite answer. Thus, it is necessary to determine long-term effects of SWCMs on runoff, sediment and their relationship on a relatively uniform slope.

Hills and mountains are widely distributed in the red soil region of southern China, covering about 60.6% of the land area. Soil erosion is one of the most severe ecological problems in the region [14, 15]. In recent years, citrus orchards have been developed extensively in this area. For example, Jiangxi Province, located in the center of red soil region in southern China, has a citrus planting area of 3331.01 km^2^, accounting for 80.32% of the total area of orchards, with an annual output of 4.10 × 10^6^t, and accounting for 91.07% of the province’s total fruit by the end of 2015. However, the steep terrain and abundant rainfall have led to serious soil erosion during the initial stage of orchard development, which will affect the sustainable development of orchards[16]. Nature based solutions concerning SWCMs were the mainstream land management strategies to offset human impacts[17]. Successful SWCMs are the key to the construction of ecologically sustainable orchards on sloping fields [18, 19]. At present, SWCMs in sloping orchards include no-tillage, cover with grass strips or mulch and agricultural intercropping, and terracing [20–22], respectively representing soil management techniques, crop and vegetation management and engineering methods. An accurate assessment of their protective effect will require evaluating the effectiveness of SWCMs over longer periods. The temporal variability in SWCM effectiveness and how this evolves over the years following the initial application are the foundation of optimizing allocation. Assessment of the long-term effect of SWCMs has focused on soil organic carbon [23], soil biochemical properties [24] and crop yield [25, 26] but no study has considered long-term effects on runoff or sediment. Most studies have focused on quantifying the effectiveness directly after application of the SWCMs using single or short-term observation data, while temporal variability and evolution of SWCMs effectiveness over time have been largely ignored [27]. With the implementation time of SWCMs increased, the changes in runoff, sediment and their relationship on slopes have not been systematically observed and analyzed.

In this study, runoff plots in the red soil region received four SWCMs: Clean-tillage (CT), grass strip (GS), agricultural intercropping (IC) and level terrace (LT). Based on data of runoff and sediment yield in 2001–2015 under natural rainfall, the variation trend of sediment and its relationship with runoff for different SWCMs in the sloping orchard were analyzed. Specific objectives were to (1) assess the long-term impact of different SWCMs on soil and water loss in this hilly red soil region and to provide some effective technologies for orchard protective practices and (2) analyze how to change the runoff–sediment relationship on the slope surface of each treated with the four different SWCMs. The results generated will provide information for the evaluation and implementation of long-term SWCMs to control soil erosion.

## Materials and methods

### Study area

**Fig 1:**
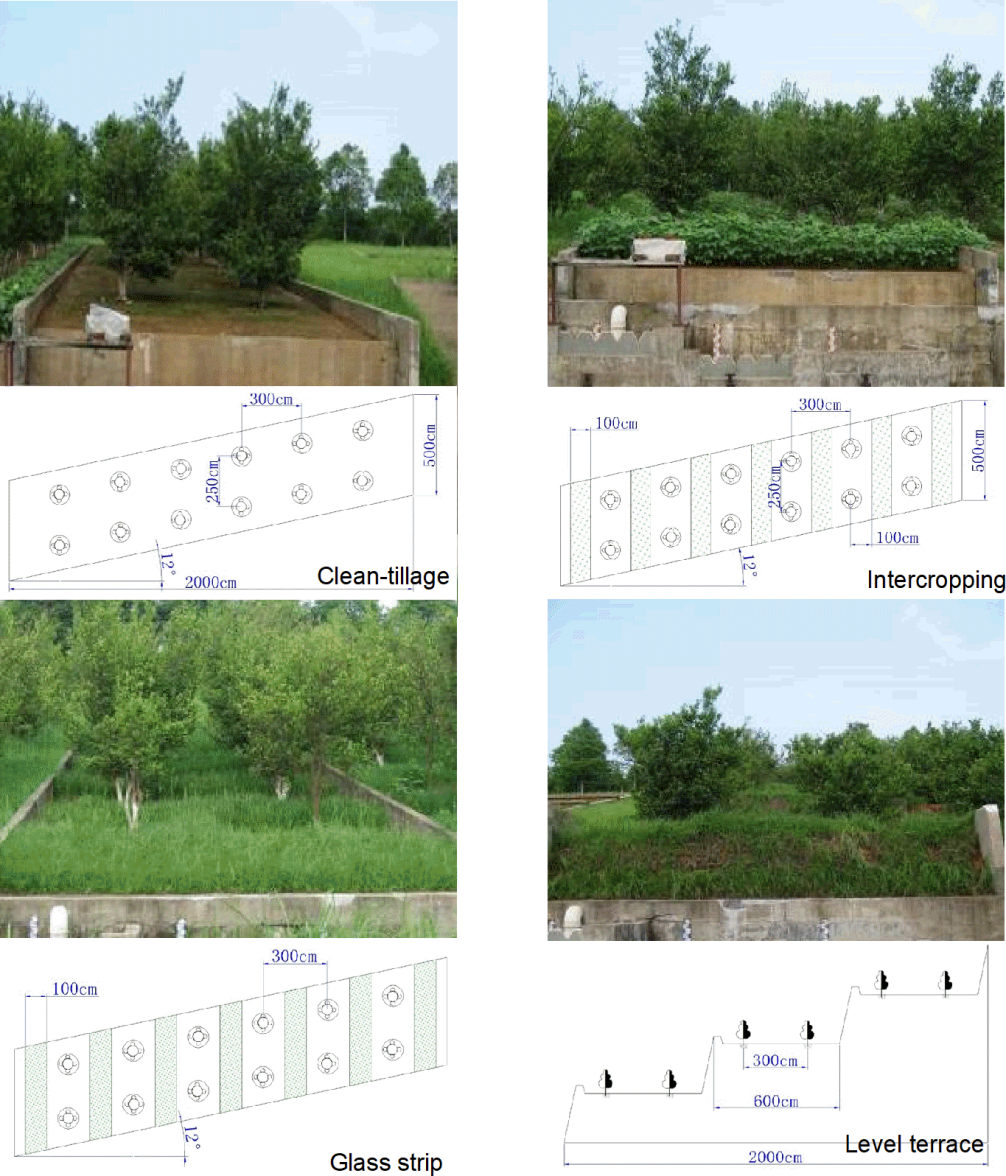
Runoff plots of SWCMs in the experimental area. The bottom half of each actual picture is the schematic diagram.

The study was conducted at the Jiangxi Eco-Science Park of soil and water conservation (29°16′–29°17′N and 115°42′–115°43′E; Fig 1), located in Dean County of northern Poyang Lake Basin. Altitude in the area is in the range of 30–90 m above sea level. The area is characterized by a subtropical humid monsoon climate zone and a mean annual precipitation of 1393.74 mm for 2001–2015. The seasonal distribution of precipitation is very uneven, with rainfall concentrated in April–September, with high intensity. The annual mean air temperature is 16.8°C, with the highest monthly temperature in July and the lowest in January. The region has approximately 245–260 frost-free days annually. The red soil in this region was primarily produced from the weathering of Quaternary sediments and soil texture is silty loam. The soil water holding capacity is weak, and the effective soil water storage capacity is small.

### Plots and measurements

Four vertical projection 20-m-long × 5-m-wide runoff plots with 12° slope were installed in 2000 on bare wasteland, with a horizontal projection area of 100 m^2^ (Fig 1). Twelve citrus trees (2 years old) were planted in each experimental plot in six rows along the slope, with two plants per row. Within-row spacing was 3 m and between-row spacing was 2.5 m. In the fourth year after the planting, the citrus trees had been mature and began to bear fruit. The experiment had four treatments under citrus trees: CT, GS, IC and LT (Table 1). CT, the control group, the soil was frequently tilled to remove as many weeds as possible. GS, Bahia grasses (*Paspalum notatu*) were interplanted along the contour lines, whose bandwidth was 1.0 m and strip spacing was 1.0 m. IC, soybeans and *Raphanus sativus* were alternate interplanted in mid-April and mid-August, respectively. LT, terraces were builtwith the slope divided into three levels, and the length and width of the surface were 6 m and 5 m, respectively. To prevent surface runoff from entering and leaving the site, the surrounding area was covered with concrete blocks, which were 30 cm above the surface and 45 cm deep underground. To test the surface flow and sediment on the red soil following different conservation measures, runoff storage containers were set in the bottom of the plots. The container volume for the surface flow was 3 m^3^ by the five-hole shunt method. Each runoff collection pool wall was fitted with an enamel water-level gauge.

**Table 1:** Design of runoff plots for different conservation measures under citrus trees.

Plot	Treatment	Surface structure
Clean-tillage	Weed control only, undisturbed land surface	Bare surface
Grass strip	Contour planting *Paspalum notatu* and the belt width is 1.0m	Grass belt
Intercropping	Soybeans and *Raphanus sativus* rotation along contour lines	Crop belt
Level terrace	Bare surface, planting *Paspalum notatu* on the terrace wall	Level terrace

### Experimental observation

According to test specifications of soil and water conservation (SD239-87 and SL419-2007) issued by the Ministry of Water Resources of China, runoff and sediment tests were carried out after each natural rainfall event during 2001–2015. The rainfall data in the test site were collected continuously by SL3-1 automatic tipping-bucket rain gauge (Shanghai Meteorological Instrument Factory Co. Ltd., China). Surface runoff was calculated from the readings of the water-level gauge in the runoff pool and the pre-established water level and volume formula. After rainfall, the runoff was stirred evenly and water samples were collected, filtered and dried to determine the sediment content, and then the total amount of erosion sediment was calculated according to runoff.

### Methods and statistical analysis

#### Effects of SWCMs on runoff and sediment

In order to eliminate the influence of rainfall change, the concepts of soil erosion modulus (*SEM*), runoff coefficient (*RC*) and rainfall erosivity coefficient (*REC*) were introduced to express the effects of SWCMs on runoff and sediment. The value of *RC*/*REC* is the ratio of the total runoff/sediment to the corresponding precipitation at any time in a given area. The *RC* and *REC* show how much precipitation was converted into surface runoff and erosion sediment, which only reflects the influence of natural geographical elements on runoff and sediment, excluding natural rainfall factors. Their formulae are as follows:

$$RC=\frac{R}{P}$$ (1)

$$REC=\frac{M}{P}$$ (2)

$$SEM=\frac{T}{A}$$ (3)

where *R* is runoff depth (mm), *M* is sediment load (kg), *P* is precipitation depth (mm), *T* is the total load of soil erosion for one year (t·a^-1^), *A* is experimental plot area (km^2^). The experimental data were subjected to analysis of variance (ANOVA) (LSD method) and fit with time using the SPSS 19.0 software package for Windows.

#### Mann–Kendall trend analysis

The Mann–Kendall rank correlation is a non-parametric statistical test, which is useful to assess temporal trends in hydro-meteorological data [28, 29]. This was applied to detect the runoff discharge and sediment load trends in the present study. The Mann–Kendall test statistic is given by:

$$S=\sum_{k=1}^{n-1}\sum_{j=k+1}^{n}Sgn(X_{j}-X_{k})$$ (4)

where *n* is the dataset record length; *X*_*j*_ and *X*_*k*_ are the sequential data values in *j* and *k* (*j* > *k*), respectively; and *Sgn*(*X*_*j*_−*X*_*k*_) is the sign function:

$$Sgn(X_{j}-X_{k})=\{\begin{array}{c} +1\\ 0\\ -1 \end{array}\begin{array}{c} (X_{j}-X_{k})>0\\ (X_{j}-X_{k})=0\\ (X_{j}-X_{k})<0 \end{array}$$ (5)

The variance (*Var*) was computed using Eq (6):

$$Var(S)=\frac{n(n-1)(2n+5)}{18}$$ (6)

In cases where the sample size *n* > 10, the Mann–Kendall test rank correlation coefficient *Z* was calculated using Eq (7):

$$Z=\{\begin{array}{c} \frac{S-1}{\sqrt{Var(s)}}\\ 0\\ \frac{S+1}{\sqrt{Var(s)}} \end{array}\begin{array}{c} S>0\\ S=0\\ S<0 \end{array}$$ (7)

The standardised *Z* statistics follow a normal standardised distribution. A positive *Z* value indicates an upward or increasing trend with time, and a negative value indicates a downward or decreasing trend. When |*Z*| ≥1.645, the trend is significant at a confidence level of 95% (one-sided test) [30].

#### Variation diagnosis of runoff and sediment relations

The relationship between runoff and sediment can be expressed by power function:

$$Ms=a\times R^{b}$$ (8)

where *Ms* is the sediment load (t·km^-2^), *R* is runoff discharge (mm), a, b are the undetermined coefficients.

There was a linear relationship between the logarithm of *Ms* and *R* (ln*Ms* and ln*R*), and the correlation coefficient can be used to describe the change of runoff and sediment relations. To assess the temporal variability in the runoff–sediment relationship, moving correlation coefficients of ln*Ms* and ln*R* were calculated for 24-month windows using the software package OriginPro 2015.

Moving correlation coefficient method had certain empirical experience in determining the variation point and need to be verified by other methods. Double mass curve (DMC) analysis is widely used to investigate the relationship between the accumulation of sediment and runoff [31]. A DMC of the runoff–sediment relationship is a plot of cumulative values of runoff volume against the cumulative sediment load during the same period. The theory behind DMC is that plotting cumulative sediment against runoff will form a straight line, with the slope representing the constant of proportionality between sediment and runoff. A break in slope indicates a change in the constant of proportionality. In this work, the DMC method was used to diagnose variation in the runoff–sediment relationship for each experiment plot.

## Results

### Rainfall variation

**Fig 2:**
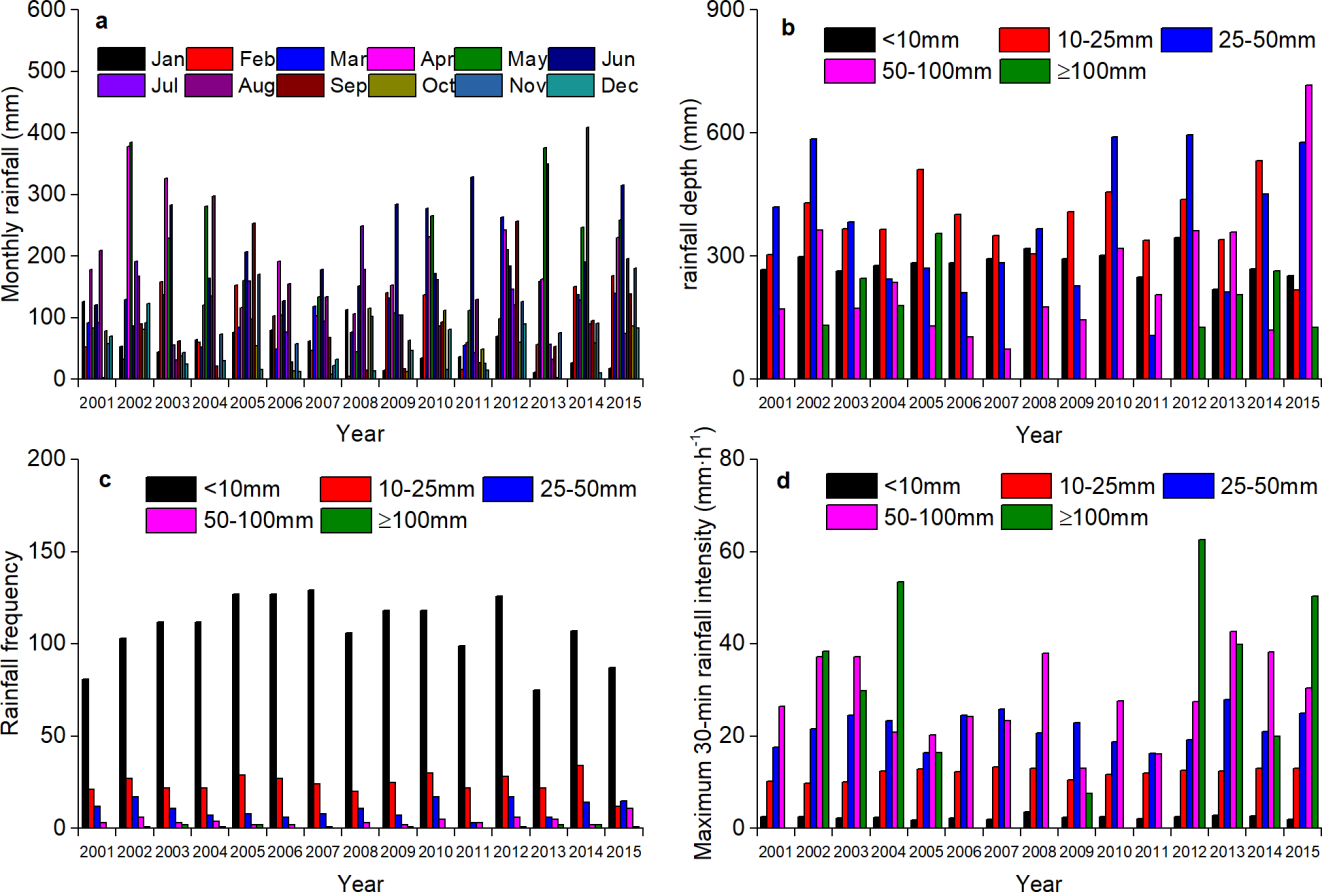
Characteristics of rainfall distribution from 2001 to 2015. (a) is Monthly distribution, (b) is the depth of rainfall grade, (c) is the frequency of rainfall grade (d) is the maximum 30-min intensity of rainfall grade.

Rainfall is a key factor affecting soil erosion in the low hilly red soil area. Therefore, understanding the variation characteristics of rainfall plays an important role in distinguishing the effectiveness of SWCMs. The annual rainfall in the experimental area was abundant during the period 2001–2015 (Fig 2), but there was a big difference between years. The maximum rainfall was 1889.7 mm (2015), and the minimum was 898.5 mm (2011), the inter-annual coefficient of variation was 0.23. The distribution of rainfall had a strong influence on the runoff and sediment of hillslopes. June had the greatest average monthly rainfall and December had the least; and the ratio between them was 4.85. The biggest variability of rainfall was for September with a coefficient of variation (CV) of 0.98, and the smallest CV was 0.39 for June. April–June is the main flood season in the study area, accounting for 25.83–42.83% of the total annual rainfall. The rainfall distribution was very uneven during the year. The rainfall events were divided into five rainfall grades by the amount of rainfall, which were less than 10mm, 10-25mm, 25-50mm, 50-100mm, and more than 100mm. The statistical results showed that, although the frequency of the rainfall events less than 10mm was 73.22%, the rainfall was only 20.09% of the total rainfall, and the maximum 30-minute intensity was the smallest, less than 3.68 mm·h^-1^. The frequency of rainfall events more than 25mm was only 10.35%, but rainfall accounted for 52.32% of total rainfall, and maximum 30-min rainfall intensity was more than 7.6 mm·h^-1^.

For the 15-year period 2001–2015, the Mann–Kendall trend test coefficient *Z* of annual rainfall was 1.089, showing an upward trend, but it was not significant (*p* < 0.05). The *Z* values of rainfall in January and August were -2.08 and -1.19 respectively, showing a decreasing trend. The *Z* values of March, June and September were 1.58, 2.47 and 1.48 respectively, showing an upward trend, and there was no obvious change in the other months. The frequency of more than 50mm precipitation events in summer (June-July-August) showed a significant increase, which contributed greatly to the increase in annual precipitation. Rainfall erosivity is the potential soil erosion due to rainfall [32, 33]. It is an objective index to evaluate soil separation and transportation caused by rainfall. The average annual rainfall erosivity was 5493.52 MJ·mm·hm^–2^·h^–1^ calculated by monthly rainfall [34], of which during April–June accounted for 49.29%. Similar to annual rainfall, annual rainfall erosivity also showed no significant upward trend according to the Mann–Kendall trend test.

### Effectiveness of SWCMs in reducing runoff and sediment

#### Rainfall event-induced runoff and sediment

Previous research suggested that runoff and sediment yields are strongly event-driven [35], and therefore water erosion was determined on the basis of rainfall events. Rainfall was considered to belong to one event if separated from the next rainfall by at least two no-rain days [36]. Compared with CT, the IC, GS and LT treatment all functioned well in reducing runoff and sediment loss for different individual rainfall grades (Table 2). With the increase of rainfall level, the benefits of the SWCMs increased. The effect of SWCMs on sediment reduction was greater than on runoff reduction, indicating that conservation measures not only reduced sediment by water reduction, but also changed runoff–sediment relationships. One-way ANOVA showed that, for rainfall < 25 mm, the runoff and sediment of the three SWCMs significantly differed from those for CT, but there were no significant difference among them.

**Table 2:** Water erosion in different individual rainfall grades for the four SWCM treatments.

Index	Rainfall (mm)	Clean-tillage	Grass strips	Intercropping	Level terrace
Runoff (mm)	≥100	37.42 ± 254.82a	2.21 ± 0.20b	18.13 ± 158.09ab	2.97 ± 0.21b
	≥50	11.38 ± 17.80a	1.02 ± 0.04b	3.96 ± 2.81ab	1.55 ± 0.04b
	≥25	4.40 ± 2.39a	0.51 ± 0.01b	1.64 ± 0.85b	0.77 ± 0.01b
	≥10	1.25 ± 0.28a	0.22 ± 0.00b	0.51 ± 0.01b	0.39 ± 0.00b
Sediment (t·km^–2^)	≥100	259.29 ± 24043.70a	0.72 ± 0.03b	133.96 ± 14474.44ab	0.91 ± 0.14b
	≥50	45.73 ± 1238.96a	0.31 ± 0.00b	21.36 ± 676.56ab	0.42 ± 0.01b
	≥25	44.49 ± 1813.33a	1.01 ± 9.61b	25.2 ± 1281.04b	0.36 ± 0.14b
	≥10	2.70 ± 6.61a	0.09 ± 0.00b	0.67 ± 0.18b	0.15 ± 0.02b

Values are means ± standard deviation and the different small letters in the same row indicate a significant difference between four SWCM treatments (LSD test, *p* < 0.05).

With heavy rain, the soil and water conservation of the IC was not significant. The IC sloping soil was disturbed by artificial tillage or harvesting activities, and heavy rain produced severe soil erosion. The rainfall of 204 mm during 13–15 August 2004 generated 1721 t·km^–2^ of soil erosion from the IC experimental area, and the erosion intensity exceeded 1251 t·km^–2^. This was because the period immediately followed the harvest of soybean. In another rainfall event of 28.3 mm on 15 September 2010, the average rain intensity was 42.5 mm·h^–1^ and the IC plot produced about 297 t·km^–2^ of erosion sediment, which was greater than the 241 t·km^–2^ of the CT plot. This was because the IC area had white radish growing but which was still in the seedling stage.

#### Annual rainfall induced runoff and sediment

**Fig 3:**
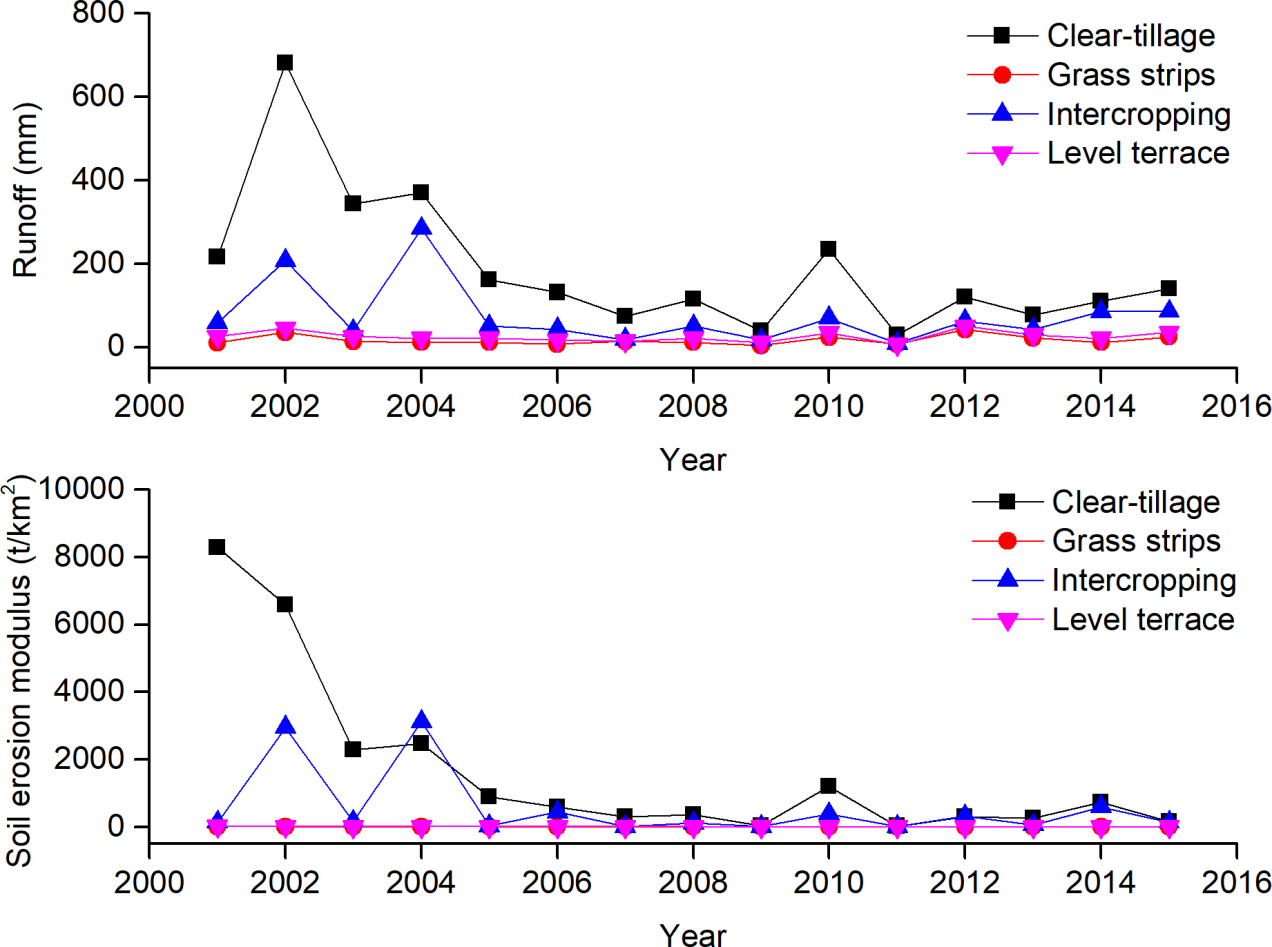
Annual runoff and sediment yield for the four treatments. CT: Clean-tillage, GS: Grass strips, IC: Intercropping, LT: Level terrace.

When GS was applied under citrus trees, the runoff and sediment decreased significantly compared with CT, and the inter-annual variability also decreased (Fig 3). Annual runoff and sediment yield of IC measures were 74.72 ± 74.47 mm (mean ± SD) and 570.3 ± 1018.92 t·km^–2^·a^–1^, respectively, and corresponding annual CVs were 1.00 and 1.79. The yield and variation of runoff and sediment by IC measures were higher than by the other SWCMs, but lower than by CT slopes. The slope erosion amounts for GS and LT were less than 500 t·km^–2^·a^–1^, the threshold for acceptable erosion in the red soil area. As for the rainfall events, one-way ANOVA showed that the runoff and sediment of SWCMs significantly differed from those for CT, but there were no significant differences among the three SWCMs. The average annual runoff and sediment of IC were higher than LT and GS, due to severe erosion by individual rainstorms. Under the same conditions, runoff and sediment yield are closely related to surface roughness [37]. Because their surface structures had the same contour bands, and therefore the average surface roughness of the three SWCMs was approximately the same, but was greater than CT.

To study the differences of *RC* and *REC* between four SWCMs in the different growth periods, 2001–2015 was divided into two periods for comparison: 2001–2004 and 2005–2015. In the first four years, the *RC* and *REC* values of the CT treatment were large, while the GS, IC and LT treatments were relatively small (Table 3). With the increase of time, the *RC* and *REC* values of each treatment decreased to very low level, and the IC reduction was the most acute. Although *RC* and *REC* of CT during the fruit period were still many times larger than GS, IC and LT, their absolute data were very small, the soil erosion intensity had been reduced below the allowable amount, and the rate of soil erosion become stable gradually. Thus, the first 4 years was the key period to prevent soil erosion in the citrus orchards.

**Table 3:** Annual runoff coefficient (*RC*) and rainfall erosivity coefficient (*REC*) of the four treatments in the different periods.

Index	Period	Clean-tillage	Grass strips	Intercropping	Level terrace
*RC*	2001–2004	0.27±0.08	0.01±0.01	0.1±0.09	0.02±0.00
	2005–2015	0.08±0.04	0.01±0.01	0.03±0.01	0.02±0.01
*REC*	2001–2004	3.56±2.53	0.005±0.00	1.07±1.14	0.009±0.01
	2005–2015	0.32±0.24	0.003±0.00	0.14±0.15	0.004±0.00

### Temporal dynamic variability and trends in the effectiveness of SWCMs

The Mann–Kendall trend test showed that *RC*, sediment content (*SC*) and *REC* in the CT plot had significant decreasing trends from the beginning (Table 4), but soil erosion during heavy rainfall was still very serious (Table 1). The runoff (*R* and *RC*) of GS and LT plots changed little after consecutive years of SWCMs application, but the sediment erosion modulus (*SEM*), *SC* and *REC* decreased significantly (*p* < 0.05). Both the runoff and the sediment of the IC plot showed no significant decreasing trend, but the sediment reduction trend was more obvious.

**Table 4:** Mann–Kendall trend analysis for runoff (*R*), runoff coefficient (*RC*), sediment erosion modulus (*SEM*), average sediment content (*ASC*) and rainfall erosivity coefficient (*REC*).

Index	Clean-tillage	Grass strips	Intercropping	Level terrace
*R*	-2.78**	0.35	-0.01	-0.20
*RC*	-2.77**	0.00	-0.79	-0.40
*SEM*	-3.07**	-1.98*	-0.79	-3.17**
*SC*	-2.77**	-1.88*	-0.69	-1.78*
*REC*	-2.97**	-2.47**	**-1.09**	-3.07**

Symbol * and ** indicate significance at 0.05 and 0.01, respectively.

**Fig 4:**
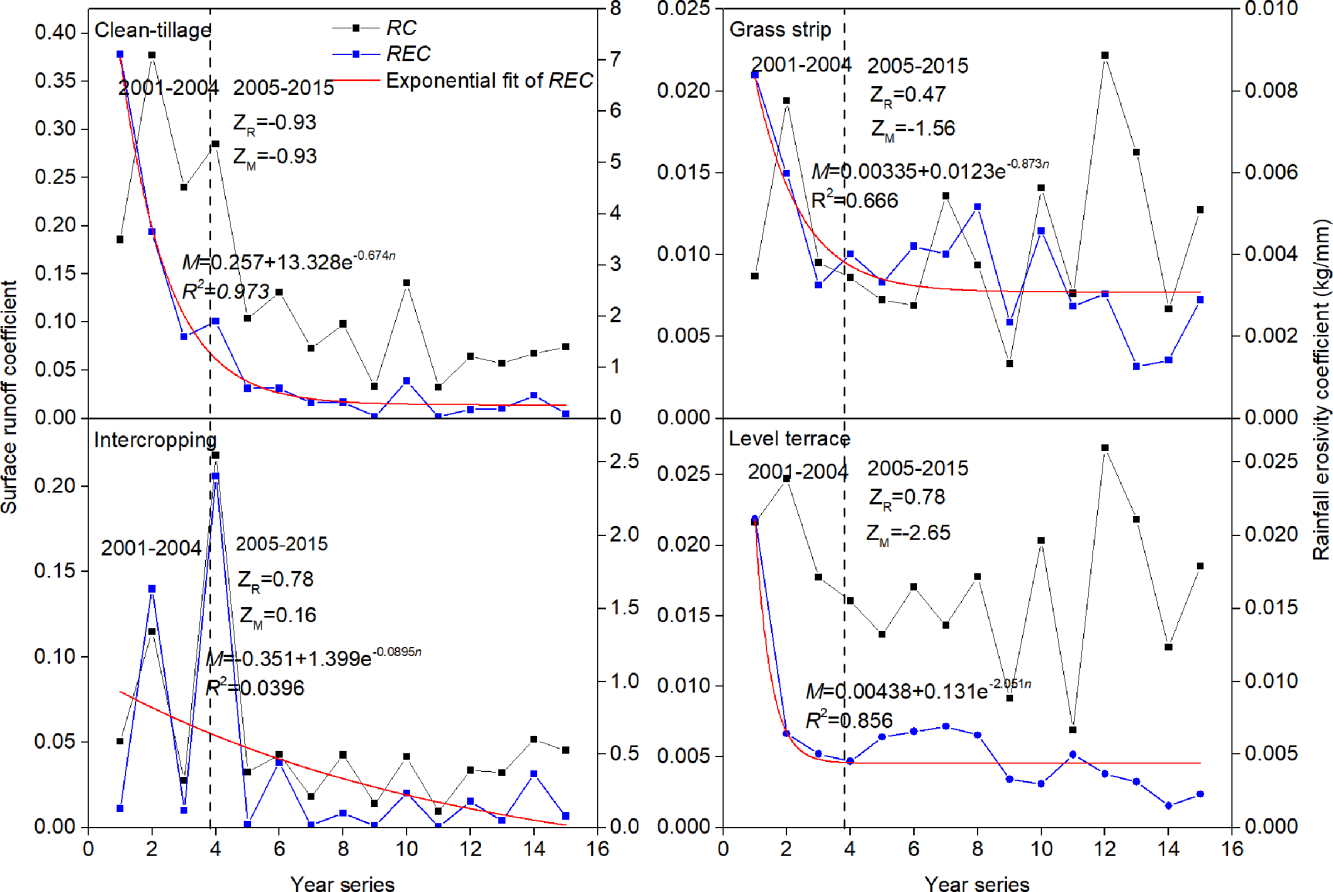
Annual runoff coefficient (*RC*) and rainfall erosivity coefficient (*REC*) for the SWCMs experimental plots during 2001–2015. Z_R_ and Z_M_: Mann–Kendall rank correlation of *RC* and *REC* during 2005–2015, respectively.

The decreases in *REC* with time were fitted with curves (Fig 4). Except for IC, the *REC* of other plots showed significant exponential functions with time. With the increase of time, the *REC* of each plot tended to have a fixed value of– 0.257, 0.00335 and 0.00438t t·km^–2^·mm^–1^ for CT, GS and LT, respectively.

The vegetation coverage of the citrus orchards improved with the tree growth. The CT measures showed that the soil and water conservation effect of citrus trees gradually strengthened in the first 4 years, and the relationship between runoff and sediment had mutated at this time, and then maintained relatively stable. This was consistent with the development of fruit trees that had been mature in 2004. Therefore, the changes of *RC* and *REC* before 2004 can be attributed to tree growth and SWCMs, but after 2004 they can be attributed to SWCMs alone. As showed in Fig 4, the effect of GS and LT treatment on *REC* decreased markedly, and that on *RC* showed a rising trend but was not significant with time. The effect of IC treatment on *RC* and *REC* showed a slight upward trend. It can be inferred that the benefit of these techniques to reduce runoff was independent of the time after SWCM application, but was increasingly important for sediment reduction. The effect of sediment reduction by citrus trees, grass strip and level terrace could be divided into rapid growth and stable periods with time.

### Runoff–sediment relationship and its variability analysis

**Fig 5:**
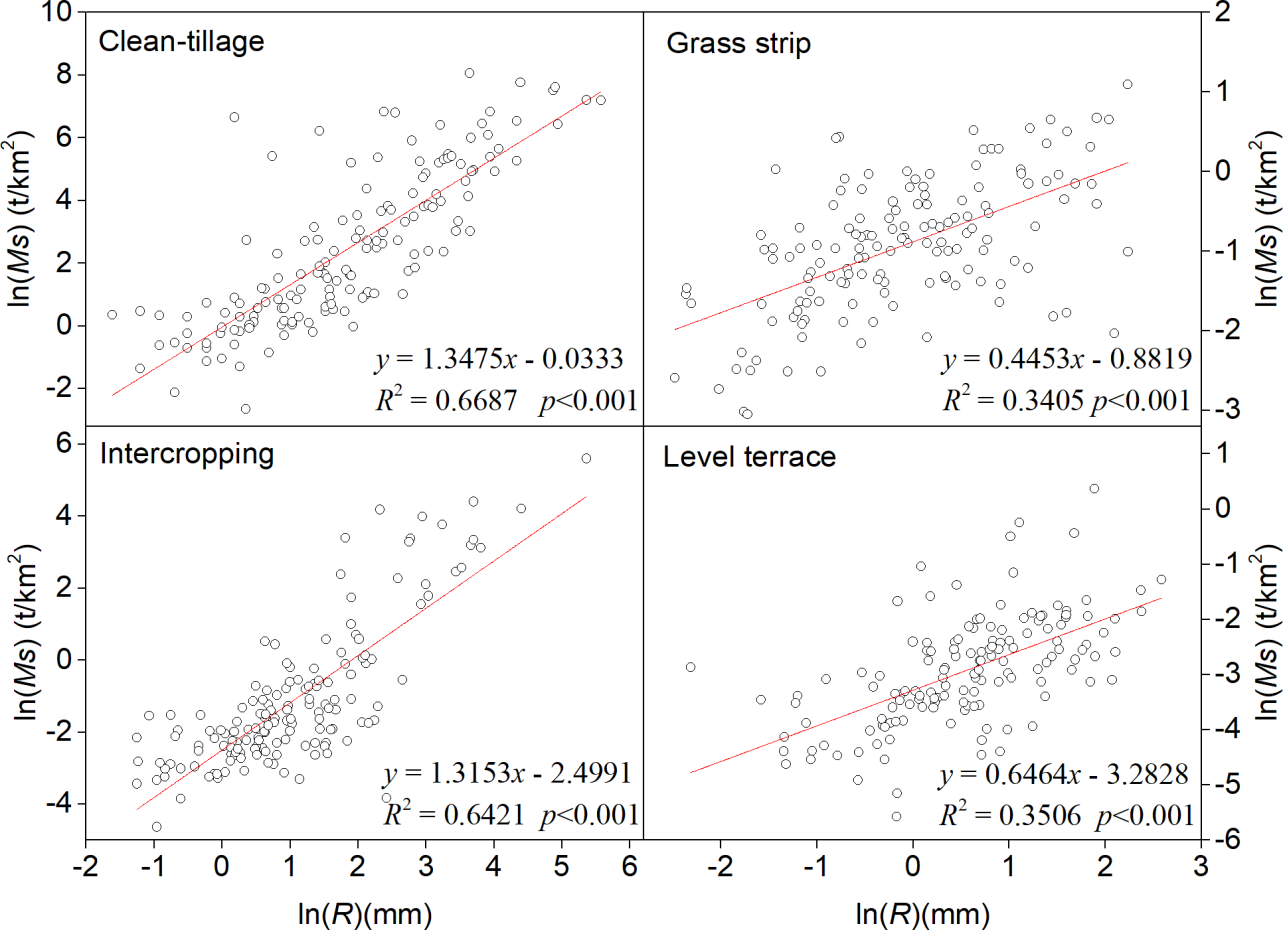
Relationship between runoff amount and sediment yield at the monthly scale.

The statistical results showed that the monthly runoff and sediment of each orchard plot have good correlation (*p* < 0.05), but the correlation between annual sediment load and runoff discharge was not clear (*p* > 0.05). Furthermore, monthly runoff had a power function relationship with sediment load (Fig 5). The slope of the fitting line of CT treatment is the largest and GS treatment is the smallest. The slope of the fitted line is larger, the sediment-carrying capacity and soil erodibility of the runoff is stronger [38]. It can be inferred that the correlation coefficient shows the effect of SWCMs on sediment reduction.

**Fig 6:**
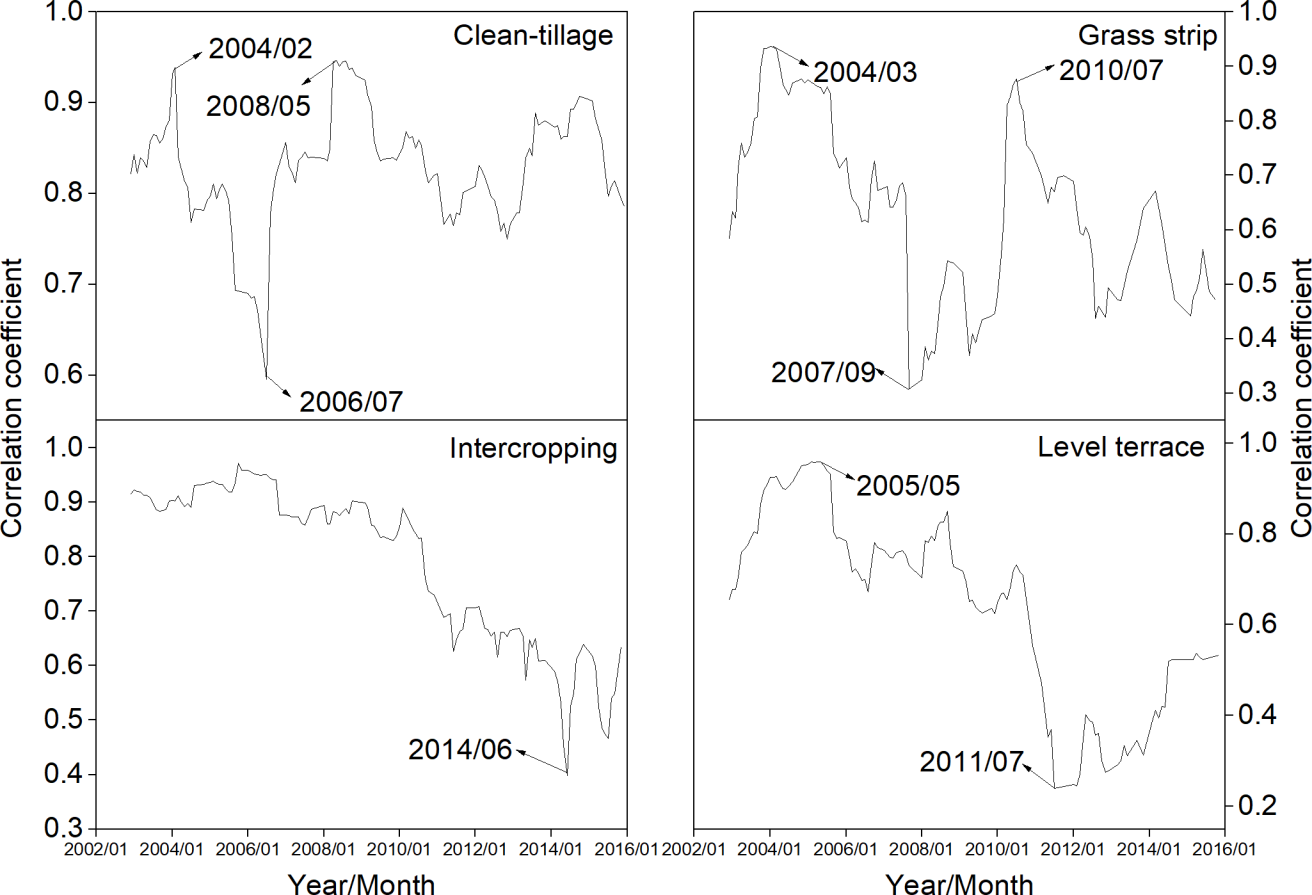
Variation detection of relationship between runoff and sediment based on moving correlation analysis.

Fig 6 represented the variation of runoff and sediment relations in four test plots by moving correlation coefficient method. The relationship between runoff and sediment in each experimental plot had mutated in 2004–2005, and then the correlation coefficient between runoff and sediment decreased, especially for IC treatment. It can be deduced that the relationship between runoff and sediment becomes weaker over time. The runoff and sediment relations of GS and LT plots had mutated again in 2010–2011.

**Fig 7:**
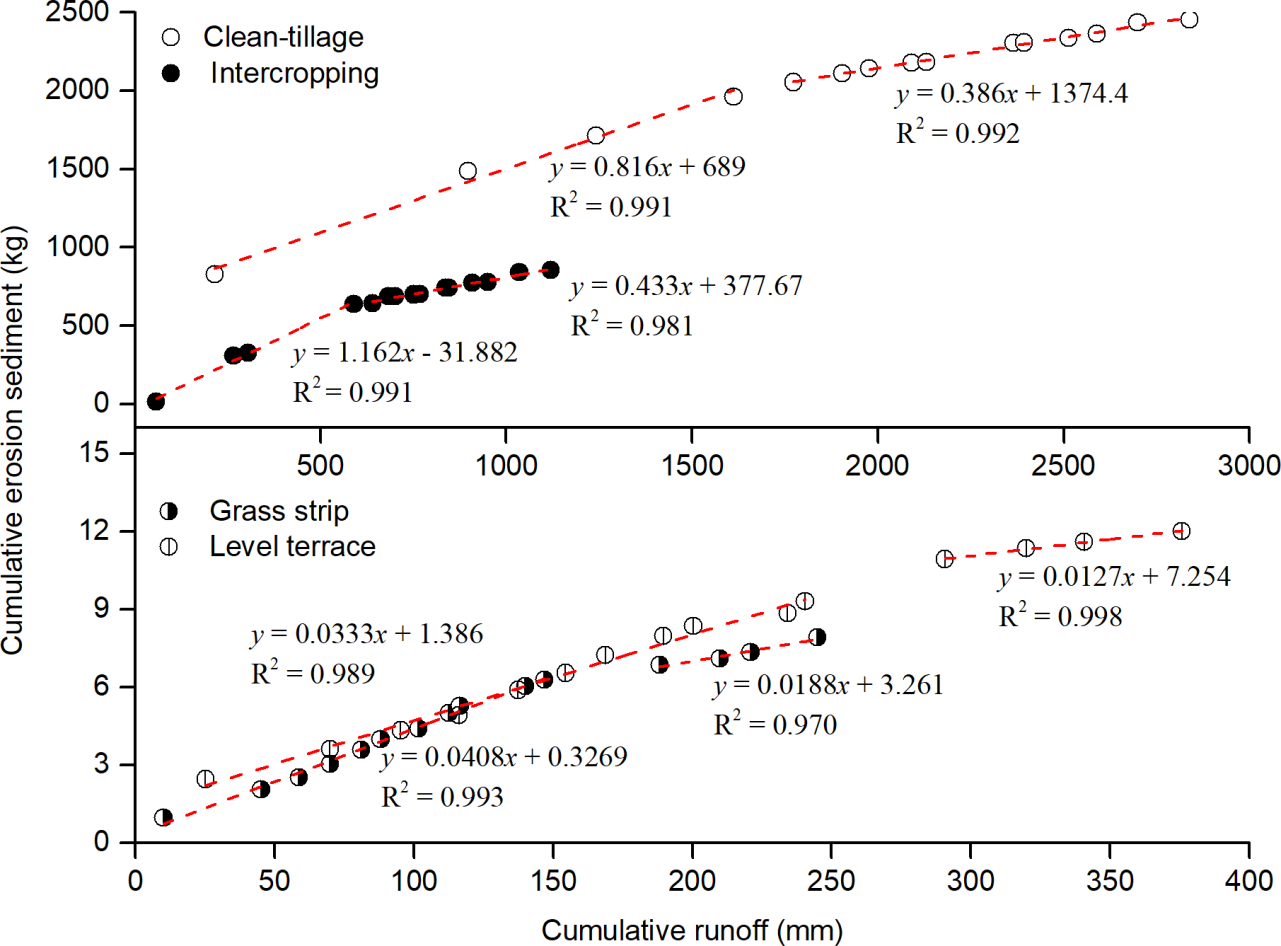
Double accumulation curves of runoff and sediment on different plots.

In order to further determine the variation of the relationship between runoff and sediment, the DMCs method was used to diagnose the change (Fig 7). The slope of DMCs for the CT and IC measures declined at about 4 years, and for the GS and LT measures at about 10 years. This indicated that the unit runoff sediment transport capacity decreased significantly, and that the runoff–sediment relationship had changed significantly. The time of significant changes in the runoff–sediment relationship for CT and IC are longer than GS and LT. The reason was that the sediment reduction effects of CT and IC measures were not significant at the initial stage, and would take a short time to make significant changes, while the effects of GS and LT measures were very noticeable and would take a long time to make significant changes.

**Fig 8:**
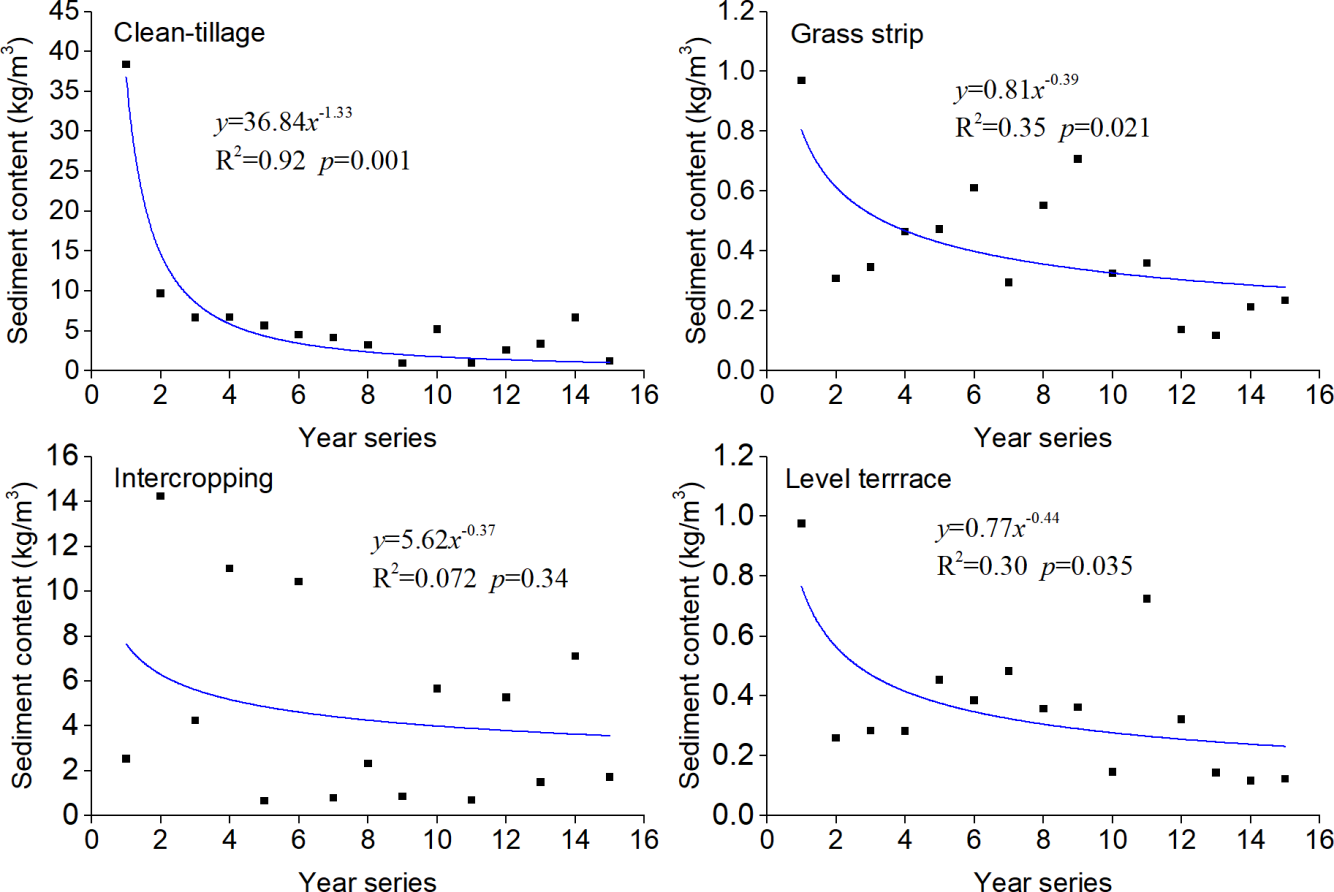
Change of sediment content with time. The first year series is 2001.

Sediment content is an important index to describe the runoff–sediment relationship, which is the sediment load of per unit runoff. SWCMs significantly reduced the sediment concentration in rainfall runoff over time–the downward trends of CT, GS and LT plots were significant (*p* < 0.05). The variation curves of sediment concentration with time had an ‘L’ form (Fig 8). This indicated that the decrease process in annual SC could be divided into two: rapid descent and relatively stability stages. With the passage of time, the effect of SWCMs on sediment gradually increased, but the effect on runoff was little changed (Table 3) and the sediment yield per unit runoff decreased. The decrease of sediment discharge on the slope surface was significantly larger than the runoff decrease, and the water–sediment relationship changed greatly. Because of the relative fixity of artificial tillage activities and the high randomness of rainfall, the relationship between runoff and sediment for IC was more complex and showed strong randomness.

## Discussion

### Soil and water conservation strategy in orchard

High erosion rates had been observed in new citrus plantations due to intense tillage and a lack of vegetative cover in the test area. Tables 2 and 3 showed the importance of soil and water conservation in the early stage of citrus orchards. Work by de Graaff et al.[39]showed that a crucial aspect for a continued adoption of SWCMs is their perceived profitability. The establishment of complex SWCMs structures can be somewhat compensated with the plantation of crops and/or fodder grasses. The IC and GS technologies were designed according to this rule in orchards. In the juvenile stage of fruit trees, the orchard vegetation coverage was low and IC effectively increased the surface coverage to prevent soil erosion, and would also provide certain economic benefits. However, due to the growing periodicity of crops, the change of surface coverage in the IC area was considerable, leading to a great fluctuation in sediment loss between years, and it was more sensitive to rainfall intensity and the duration response to single rainfall events. The IC only reduced soil erosion for small rainfall events, but the benefits in the event of heavy rain decreased significantly–thus simple IC is not the best choice to reduce soil and water loss in the hilly red soil region. The GS measure not only was an effective way to reduce soil erosion in the sloping orchard, but can also improve soil structure and increase the soil surface organic matter and effective reservoir capacity [18, 40], which helps increase infiltration and reduce the effect of seasonal drought [41]. Because there was no man-made tillage disturbance, the benefit of interplanting grass (i.e. GS) was greater than that of crops (i.e. IC). In this study, GS had a greater soil conservation effect than LT. Engineering measures, such as terraced fields, were also effective measures for soil and water conservation, but their economic costs are very high relative to vegetation measures.

### Variation of runoff and sediment reduction effect by SWCMs

The runoff and sediment reduction effects of SWCMs are usually calculated by comparison with control measures [42, 43]. However, due to heterogeneity of soil and topography, the compared conditions (controls) are difficult to keep strictly consistent. Furthermore, it cannot eliminate the influence of rainfall change. The change of runoff and sediment production on the slope was affected by natural rainfall and conservation measures. For the slope-scale, both the annual *RC* and *REC* excluded the natural rainfall factor, and their changes were only caused by SWCMs. Therefore, they directly reflect the dynamic changes for effects of SWCMs. It should be noted that the changes of *RC* and *REC* only reflect SWCMs’ reduction of the strength of runoff and sediment, but do not show the amount of runoff and sediment reduction.

Ran et al. [44] and Zheng et al. [45] pointed out that the effect of appropriate vegetation measures on runoff and sediment reduction will become increasingly significant with time. In the runoff plot, SWCMs had a good interception effect on runoff and sediment, and over time the interception effect on sediment became stronger, but it had little influence on runoff change. Maetens et al. [27] analyzed annual runoff during multiple years of application of SWCMs and found that CT and conservation tillage became much less effective in reducing annual runoff over time, but no such effect was observed for annual soil loss. The reason is likely to be increased surface sealing when surface soil was not disturbed for several years–although this is beneficial for sediment reduction, it also reduces infiltration capacity of soil and thus enhances runoff loss [39]. After SWCMs have been implemented in many watersheds, sediment loads in the basins decreased significantly, while runoff did not change significantly [46, 47]. The similar phenomenon was also observed in the Poyang Lake Basin [48, 49]. Therefore, it can be concluded that water conservation benefits of SWCMs can be considered as a fixed value, but soil conservation benefits vary with time. As noted by Xu [50] in a watershed, the sediment reduction benefit of SWCM is a non-linear change, which can be divided into three stages: (1) increasing slowly, (2) increasing rapidly and (3) remaining unchanged or even decreasing. After more than 30 years of soil erosion control in the Poyang Lake watershed, serious soil erosion still occurs in some areas [51]. It is urgent to solve this problem and this may require strengthened governance in the future. The results of this study indicate that soil erosion intensity will gradually decrease with the increase in the time of soil and water conservation. In some places, soil erosion will be reduced to a mild intensity after about 4 years as long as there is no human disturbance. Therefore, the future layout of SWCMs should consider the regularity of soil conservation benefits over time, rather than blindly expanding the soil erosion standard.

### Sediment reduction mechanism of SWCMs

The sediment concentration in the test plots decreased gradually and then remained relatively stable. As a result, the overland flow became clearer. The sediment yield was reduced mainly by decreases in high runoff and high sediment concentration conditions at the initial stage. Then, during the stable period, sediment yield was reduced due to decreases in overland flow and sediment concentration at all magnitudes of rainfall. It can be concluded that the water–sediment relationship on the slope have changed.

The change in runoff, sediment and their relationship are mainly affected by natural rainfall and SWCMs [52, 53]. In the past 15 years, the annual rainfall amount and rainfall erosivity in the experimental area increased slightly (Fig 2). The DMC theory holds that if the slope of the curve changes is not caused by rainfall, then it is caused by human factors. Therefore, SWCMs were the main reasons for the variation in water, sediment and their relationships on the slopes. At the plot scale, SWCMs not only reduced runoff and sediment, but also changed their mutual relationship. This change in relationship can be attributed to the fact that the function of sediment reduction has gradually strengthened. However, the reinforcement process was not linear–it initially increased and then remained basically stable. Therefore, the soil erosion reduction mechanism of SWCMs in the early phase is the joint function by reducing runoff and changing the relationship between runoff and sediment, and in the post-stable phase mainly by reducing surface runoff.

## Conclusions

This study estimated the long-term effectiveness of different SWCMs in reducing both overland runoff and sediment erosion. Vegetation and crop management (i.e. GS and IC) and engineering methods (i.e. LT) were more effective in reducing runoff and sediment than soil management measures (i.e. CT) on the sloping red soil of the orchard. SWCMs were generally much more effective in reducing soil loss than in reducing runoff. The effectiveness of SWCMs in sediment reduction showed marked temporal variations–increasing rapidly with time and then remaining stable, while runoff reduction was always relatively stable. The fitted curves showed that the benefits of soil conservation had non-linear variation, which could be divided into two stages: increasing rapidly and remaining unchanged. Trend analysis showed that intensity of soil erosion reached a stable state after approximately three years for LT and GS measures, and five years for CT. The risk of soil erosion was still serious following IC and CT measures, although the rate of runoff loss and soil erosion decreased rapidly over time. The first 4 years was the key period to prevent soil erosion, and then the intensity of soil erosion decreased to less than 500 t·km^–2^·a^–1^, if the surface was not disturbed. The LT and GS measures had similar effects on reducing runoff and sediment–so establishing pasture is the best protection due to economic considerations for citrus orchards on this sloping red soil.

The SWCMs did not change the rainfall–runoff relationship but did change the runoff–sediment relationship. They significantly reduced the sediment discharge per unit runoff, which was the cause for the significant change in the runoff–sediment relationship for this orchard. At the slope-scale, SWCMs reduced sediment by reducing surface runoff and changing the runoff–sediment relationship.

## Supporting information

S1 Table Fig 2 supporting data. (XLSX)

S2 Table Table 2 supporting data. (XLSX)

S3 Table Fig 3 supporting data. (XLSX)

S4 Table Fig 4 supporting data. (XLSX)

S5 Table Fig 5 supporting data. (XLSX)

S6 Table Fig 6 supporting data. (XLSX)

S7 Table Fig 7 supporting data. (XLSX)

S8 Table Fig 8 supporting data. (XLSX)
